# The Steroid Hormone 20-Hydroxyecdysone Regulates the Conjugation of Autophagy-Related Proteins 12 and 5 in a Concentration and Time-Dependent Manner to Promote Insect Midgut Programmed Cell Death

**DOI:** 10.3389/fendo.2018.00028

**Published:** 2018-02-07

**Authors:** Yong-Bo Li, Ting Yang, Jin-Xing Wang, Xiao-Fan Zhao

**Affiliations:** ^1^Shandong Provincial Key Laboratory of Animal Cells and Developmental Biology, School of Life Sciences, Shandong University, Jinan, China

**Keywords:** steroid hormone, ATG12–ATG5 conjugation, autophagy, apoptosis, programmed cell death

## Abstract

Autophagy requires the conjugation of autophagy-related protein 12 (ATG12) to autophagy-related protein 5 (ATG5) through covalent attachment. However, the signals regulating ATG12–ATG5 conjugation are unclear. The larval midgut of lepidopteran insects performs autophagy and apoptosis sequentially during the transition of larvae to pupae under regulation by the steroid hormone 20-hydroxyecdysone (20E), thus representing a model to study steroid hormone regulation of ATG12–ATG5 conjugation. In the present study, using the lepidopteran insect *Helicoverpa armigera* as a model, we report that 20E regulates the conjugation of ATG12–ATG5 in a concentration and time-dependent manner. The ATG12–ATG5 conjugate was abundant in the epidermis, midgut, and fat body during metamorphosis from the larvae to the pupae; however, the ATG12–ATG5 conjugate level decreased at the time of pupation. At low concentrations (2–5 µM) over a short time course (1–48 h), 20E promoted the conjugation of ATG12–ATG5; however, at 10 µM and 72 h, 20E repressed the conjugation of ATG12–ATG5. ATG12 was localized in the larval midgut during metamorphosis. Knockdown of *ATG12* in larvae caused death with delayed pupation, postponed the process of midgut programmed cell death (PCD), and repressed ATG8 (also called LC3-I) transformation to LC3-II and the cleavage of caspase-3; therefore, knockdown of *ATG12* in larvae blocked both autophagy and apoptosis. Knockdown of *ATG12* in *H. armigera* epidermis cell line cells also repressed 20E-induced autophagosome formation and caspase-3 activation. The results suggested that 20E plays key role in the regulation of ATG12–ATG5 conjugation in a concentration and time-dependent manner for autophagy or apoptosis, and that ATG12 is necessary by both autophagy and apoptosis during insect midgut PCD.

## Introduction

Programed cell death (PCD) includes apoptosis (type I PCD) with features of nuclear condensation and fragmentation, formation of apoptotic bodies, and caspase activation; autophagic cell death (type II PCD), with the accumulation of autophagosomes and autolysosomes, along with cell death; necrotic cell death (type III PCD), which involves cell swelling and membrane rupture (1); and ferroptosis, a non-apoptotic forms of cell death induced by the small molecule erastin (2). Autophagy is a self-protective phenomenon that plays a critical role in cell survival (3). A series of autophagy-related proteins (ATGs) participate in autophagy. Among them, ATG12 is an ubiquitin-like protein that conjugates with ATG5 to play vital roles during autophagy (4). ATG12 forms an isopeptide bond with ATG5 through activation of ATG7 and under the mediation of ATG10, finally forming the ATG12–ATG5 conjugate (5), which then associates with ATG16 to form a larger ATG12–ATG5–ATG16 complex (6). The complex functions as an ubiquitin–protein ligase (E3)-like enzyme to transfer microtubule-associated protein 1A/1B-light chain 3 (LC3 in mammals, also known as ATG8 in yeast) from ATG3 to phosphatidylethanolamine (PE) by stimulating the E2 activity of ATG3 (7). The ATG12–ATG5–ATG16 complex and ATG8/LC3 drive autophagosome membrane expansion and fusion (8). However, it is unclear how upstream factors, especially steroid hormones, regulate ATG12–ATG5 conjugation.

The insect midgut undergoes remodeling during insect metamorphosis from larvae to pupae to adults, which includes larval midgut PCD and the pupal and adult imaginal midgut formation (9). The larval midgut changes color from pale yellow to red, condenses toward the midgut lumen, and separates from the newly formed imaginal midgut, which are characteristics of midgut PCD (10). The steroid hormone 20-hydroxyecdysone (20E) promotes insect pupation, decreases the pupation time, and promotes larval midgut PCD by upregulating the expression of a set of genes (11). The titer of 20E changes constantly during development from larvae to pupae. The 20E titer is 1 µM during feeding, reaching a maximum of approximately 8.6 µM at the molt stage in the lepidopteran *Manduca sexta* (12). The change in 20E titer results in up- or downregulation of downstream genes (e.g., *HR3* and *E75*), which regulates the growth and metamorphosis of insects (13).

Autophagy plays a critical role in insect larval midgut PCD. A series of ATGs participate in autophagy in the insect midgut (14). In lepidopteran insects, cells exhibit both autophagic and apoptotic characteristics in many organs during metamorphosis from larvae to pupae (15). Autophagy precedes apoptosis during PCD in the silkworm larval midgut, and apoptosis is actually responsible for cell death and for the disappearance of larval midgut cells, whereas autophagy is a protective mechanism or an initial condition of apoptosis (16). 20E upregulates autophagic or apoptosis genes to promote autophagy or apoptosis in the *Bombyx* fat body (17, 18). A recent study demonstrated that 20E promotes the switch from autophagy to apoptosis during midgut PCD in *Helicoverpa armigera* by increasing intracellular calcium levels (19).

Therefore, the midgut of lepidopteran insects represents a suitable model to study the regulation of autophagy by steroid hormones and the relationship between autophagy and apoptosis. In this study, we report that low concentrations, a short period of 20E promote ATG12–ATG5 conjugation and high concentrations, a long period repress ATG12–ATG5 conjugation during midgut PCD in the lepidopteran insect *H. armigera*. ATG12 is necessary for both autophagy and apoptosis. The study provides new insights into the steroid hormone regulation of autophagy and apoptosis in the insect midgut during metamorphosis.

## Materials and Methods

### Cloning and Sequence Analysis of *ATG12* cDNA

The full sequence of the *ATG12* cDNA open reading frame (ORF) was cloned from a cDNA library of *H. armigera* larvae using PCR with two specific primers, *ATG12*F and *ATG12*R (Table 1). Protein translation and prediction was performed using the ExPASy software.^1^ The ORF contains 381 nucleotides and encodes a protein of 126 amino acids, giving the calculated molecular mass of 14.2 kDa. Multiple sequence alignment revealed that *H. armigera* ATG12 exhibited a high degree of identity with ATG12 proteins from other species: 83% to the ATG12-like proteins of *B. mori* and *Biston betularia*; 78% to the ATG12-like protein of *Danaus plexippus*; and 63% identity to ATG12-like protein of *Camponotus floridanus*.

**Table 1:** Primers used in the experiments.

Primer name	Sequence (5′–3′)
ATG12F	CACCGTCACCAGACCAGTTAG
ATG12R	TGATTTCCCACAATGAACCTTA
ATG12exF	TACTCAGGATCCATGGGTGACGAAAAGCAGCCT
ATG12exR	TACTCACTCGAGTCAGCCCCAAGCTTGACTTTTG
ATG12RNAiF	AAATAAATGGGTGACGAAAAGC
ATG12RNAiR	CTAACTGGTCTGGTGACGGTGC
GFPRNAiF	TGGTCCCAATTCTCGTGGAAC
GFPRNAiR	CTTGAAGTTGACCTTGATGCC
GFPF	TACTCAAGATCTCGATGAGCAAGGGCGAGGAAC
GFPR	TACTCAGCGGCCGCTCTTGTACAGCTCGTCCAT

### Recombinant Expression of *ATG12* in *Escherichia coli* and Preparation of Antiserum

Specific primers *ATG12*EXPF and *ATG12*EXPR (Table 1) were designed to amplify the *ATG12* ORF. The DNA sequence was digested with BamH I and Xho I and ligated into the expression vector pGEX-4T-1, which has a glutathione S-transferase (GST) tag (Merck, Darmstadt, Germany). The recombinant plasmid, designated pGEX-*ATG12*, was screened in DH5α competent cells. Plasmid pGEX-*ATG12* was then transformed into competent *E. coli* strain Rosseta. A single-colony transformant was inoculated into Luria–Bertani broth and the culture was shaken at 37°C overnight. The overnight culture was diluted 100-fold and inoculated in 200 mL of a fresh LB medium containing 100 µg/mL ampicillin. When the optical density (OD_600_) reached approximately 0.6, isopropyl β-D-1-thiogalactopyranoside was added to a final concentration of 0.5 mM to induce the expression of the fusion protein for 4 h at 37°C. Harvested cell pellets were suspended in phosphate-buffered saline (PBS; 140 mM NaCl, 2.7 mM KCl, 10 mM Na_2_HPO_4_, 1.8 mM KH_2_PO_4_, pH 7.4) and sonicated for 20 min in an ice bath, with a 10 s on/off cycle to prevent overheating. The total cellular proteins were then partitioned into soluble and insoluble fractions by centrifugation at 12,000 × *g* for 10 min at 4°C. SDS-PAGE was then performed to determine the molecular mass and solubility of the fusion protein GST-ATG12. The GST-ATG12 protein was expressed with a molecular mass of 41 kDa (GST 27 kDa + ATG12 14 kDa = 41 kDa) and existed primarily in the pellet, which was then purified by washing the inclusion bodies. The purified recombinant GST-ATG12 protein was then used to prepare rabbit polyclonal antibodies.

### Quantitative Real-time Reverse Transcription-PCR (qRT-PCR)

Total RNA was extracted from *H. armigera* using the Unizol Reagent according to the manufacturer’s instructions (Biostar, Shanghai, China). After determining the RNA quality by electrophoresis on a 1% agarose gel, 5 µg of RNA was reverse transcribed into cDNA. The resulting cDNAs were then used as the templates in PCR reactions. qRT-PCR was performed using SsoFast™ EvaGreen Supermix (Bio-Rad, Hercules, CA, USA), according to the manufacturer’s instructions and in a real-time thermal cycler (Bio-Rad). β-actin was amplified for internal standardization. The experiments were repeated three times, and the experimental data were analyzed statistically using Student’s *t*-test. The relative expression data from the qRT-PCR experiments were calculated using the 2^−ΔΔ^*^CT^* method (20).

### Immunoblotting (Western Blotting)

Total proteins from various tissues were extracted using Tris buffer saline (TBS, 10 mM Tris–HCl, pH 7.5; 150 mM NaCl with 1 mM phenylmethanesulfonyl fluoride) from three larvae to eliminate individual differences. The protein concentration was measured according to the Bradford method (21). Equal amounts of protein (20 µg) for each sample were subjected to SDS-PAGE. Proteins were transferred electrophoretically onto a nitrocellulose membrane. The membrane was incubated with blocking buffer (2% skim milk powder dissolved in TBS) for 1 h at room temperature. The antiserum against *Helicoverpa* ATG12 was diluted to 1:100 in blocking buffer in TBS and incubated with the membrane for 2 h at room temperature (21–25°C). After washing, the membrane was incubated with the secondary antibody (alkaline phosphatase conjugated goat anti-rabbit IgG diluted 1:10,000 in the blocking buffer) for 2.5 h at room temperature. The protein signal was visualized using 45 µL of nitroblue tetrazolium (75 mg/mL) and 35 µL of 5-bromo-4-chloro-3-indolylphosphate (50 mg/mL) (Sigma) in 10 mL of TBS in the dark at room temperature. The SDS-PAGE gel concentration was 12.5% unless otherwise stated. The protein bands on the membrane were analyzed using Quantity One software.^2^

### 20E Treatment of the *H. armigera* Epidermis Cell Line (HaEpi)

*Helicoverpa armigera* epidermis cell line cells were cultured with 5 µM 20E for 1, 6, 24, 48, or 72 h in Grace’s medium at 27°C. The same volume of dimethyl sulfoxide (DMSO) was added as a 20E solvent control. The total proteins were extracted from cells using 40 mM Tris-HCl for western blot assays.

### Immunohistochemistry

The larvae midgut was separated and treated with 4% paraformaldehyde at 4°C overnight and then gradient-dehydrated. The prepared midgut tissues were embedded in paraffin. They were then cut into 7-µm sections and adhered to gelatin-coated glass slides, before being dried at 37°C overnight. Subsequently, the slides were dewaxed, gradient-dehydrated, and digested with 20 µM proteinase K at 37°C for 10 min. The slides were then blocked in 2% BSA at 37°C for 30 min before the addition of rabbit anti-ATG12 antibodies at 4°C overnight. The slides were washed with PBS three times and then 1 µL of secondary antibody (goat anti-rabbit-Alexa Fluor 488 antibody in 10 mL blocking buffer) was added and incubation continued at 37°C for 1 h. The nuclei were stained by 4′, 6-diamidino-2-phenylindole (AnaSpec Inc., San Jose, CA, USA) for 10 min at room temperature. An Olympus BX51 fluorescence microscope (Shinjuku-ku, Tokyo, Japan) was used to observe the fluorescence on the sections.

### Hematoxylin and Eosin Staining

The histological sections were dewaxed, gradient-dehydrated, stained with hematoxylin for 10 min, washed with running water for 1 min, washed with Scott TapWater/Bluing (0.35 g NaHCO_3_ and 2 g MgSO_4_ dissolved for 1 min) for 1 min, washed with hydrochloric acid ethanol differentiation medium (concentrated hydrochloric acid mixture of 70% ethanol 1:100) for 20 s, washed with Scott TapWater/Bluing for 1 min, and washed with 1% ammonia water for 30 s. The sections were then stained with 0.5% eosin for 30 s and washed for 1 min with 1% ammonia water. The morphology of the cells in the sections was then observed under an Olympus BX51 fluorescence microscope.

### RNA Interference (RNAi) in Larvae

The primers *ATG12-RNAiF* and *ATG12-RNAiR* (Table 1) were designed to amplify the *ATG12* gene fragment to synthesize double-stranded RNA (dsRNA) according to the method of the MEGAscri RNAi Kit (Ambion Inc., Austin, TX, USA). One hundred and twenty larvae were divided into two groups of 60 larvae. One group was injected with *ATG12* dsRNA (*dsATG12*) (1 μg/larva), whereas the other was injected with *dsGFP* (1 μg/larva). The dsRNA was injected into the hemocoel of the sixth instar 6 h larva. A second injection was administered 24 h later. After 48 h, the RNA of each treatment group was extracted from three larvae for further detection. Independent experiments were conducted three times for statistical analysis.

### RNAi in the HaEpi Cell Line

The primers *ATG12*F and *ATG12*R, and *GFP*F and *GFP*R (Table 1) were used for PCR amplification of the *ATG12* and green fluorescent protein (*GFP*) gene fragments, respectively. The PCR products were purified using a PCR purification kit (Shenggong, Shanghai, China). dsRNA was synthesized using the MEGAscript™ RNAi kit (Ambion Inc., Austin, TX, USA). HaEpi cell line culturing and transfection were performed according to the methods described by Shao et al. (22). *dsGFP* was used as the control.

### Autophagosome Detection in HaEpi Cells

Preparation of rabbit polyclonal antibodies against *H. armigera* ATG8/LC3. *H. armigera* ATG8/LC3 was expressed in *E. coli* from the *pET-30a* plasmid. Two hundred micrograms of purified ATG8/LC3 in 1 mL TBS was mixed with 1 mL complete Freund’s adjuvant and injected subcutaneously into a rabbit to induce antibodies, according to a previously published method (23). ATG12 antiserum was prepared by the same method. To indicate autophagosomes in HaEpi cells directly, a DNA sequence, TATGGCAGGAAGAAGCGGAGACAGCGACGAAGA, encoding the cell-penetrating peptide TAT (24) was cloned into RFP (red fluorescence protein)-LC3 plasmid (His-TAT-RFP-LC3-His). The His-TAT-RFP-LC3-His protein was expressed in *E. coli* as a soluble protein and was purified by His tag affinity resin. His-TAT-RFP-LC3-His was able to penetrate into cells to detect LC3 puncta in HaEpi cells. TAT has no cell toxicity (25).

### Apoptosis Detection in HaEpi Cells

*Helicoverpa armigera* epidermis cell line cells were incubated with 5 µM 20E for 48 h. Anti-human, mouse, and rat caspase-3 polyclonal antibodies were used to detect the active caspase-3 band in western blots (WL01589, Wanleibio, Shen Yang, China). These antibodies recognize cleaved-caspase-3 (16, 26). The NucView™ Caspase-3 assay kit (NO. 30029 Biotium, Hayward, CA, USA) was used to detect the caspase-3 activity in HaEpi cells by immunocytochemistry, according to the manufacturer’s instructions. The principle of the staining is that the caspase-3 substrate peptide DEVD is attached to a DNA-binding dye. When the substrate is cleaved by caspase-3 in the cells, the fluorogenic DNA dye stains the nucleus. Therefore, the substrate not only detects caspase-3 but also visualizes the apoptotic nuclear morphology.^3^

### Statistical Analysis

Bars represent mean ± SD of three independent experiments. For statistical analyses, significance was determined using two-tailed paired Student’s *t*-test. Differences were considered significant at *p < 0.05 and **p < 0.01.

## Results

### The ATG12–ATG5 Conjugate Level Increases during Metamorphic Molting (MM)

**Figure 1:**
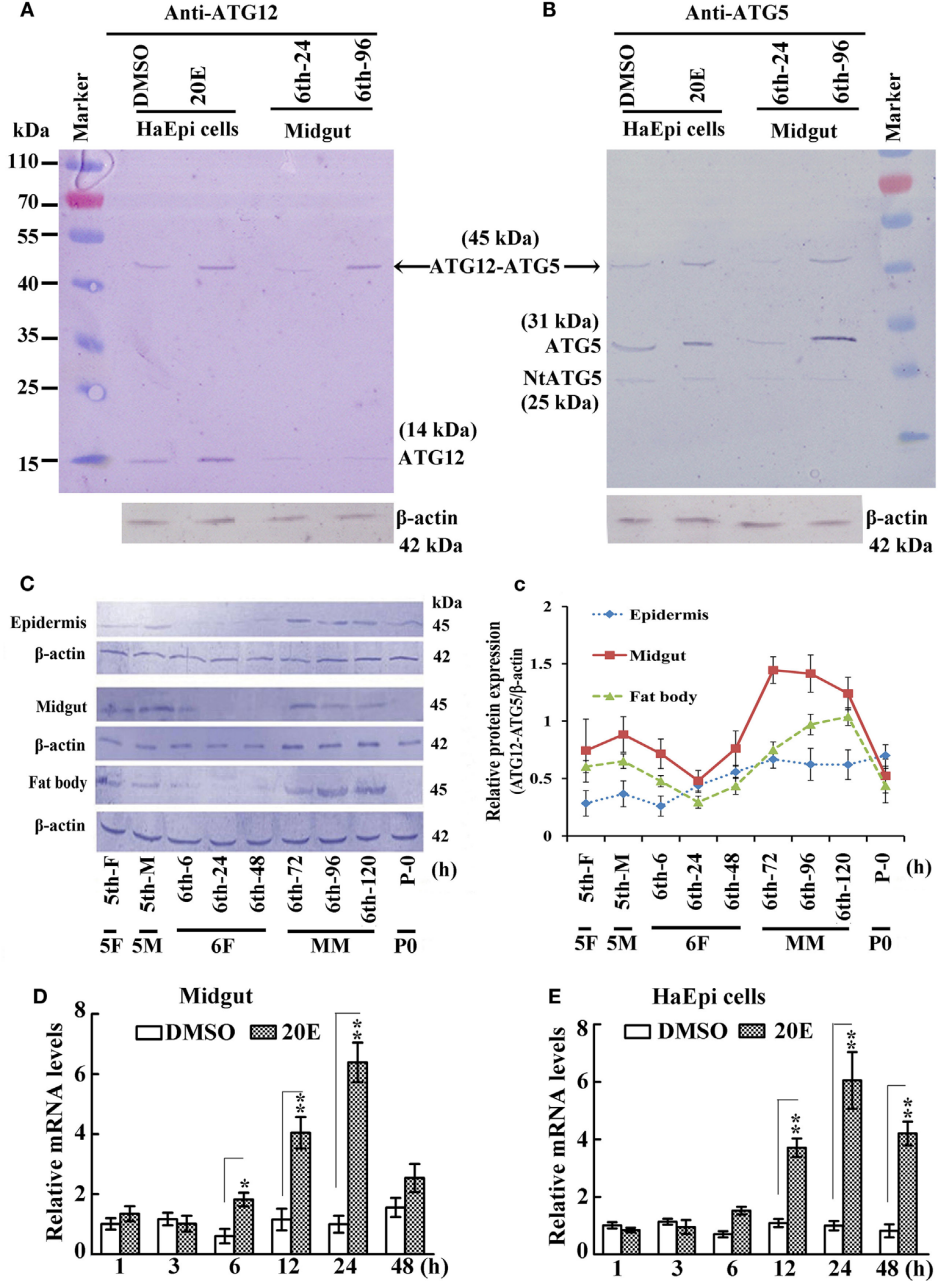
Analysis of ATG12–ATG5 conjugate levels in larval development by western blot. **(A)** Detection of ATG12 and the ATG12–ATG5 conjugate using an antibody against *Helicoverpa armigera* ATG12 in *H. armigera* epidermis cell line (HaEpi) cells and in the midgut at the indicated times of the sixth larval instar (6th-24 h, 6th-96 h). 20-Hydroxyecdysone (20E) treatment was 5 µM final concentration for 24 h. **(B)** Detection of ATG5 and ATG12–ATG5 conjugate using an antibody against *H. armigera* ATG5 in HaEpi cells and the midgut as in **(A)**. Both antibodies were prepared by our laboratory. **(C)** Developmental profile of the ATG12–ATG5 conjugate in *H. armigera* tissues; detected by anti-ATG12 antibody; an antibody against *H. armigera* β-actin was used as a loading control. 5th-F, 5th instar 12 h feeding larvae; 5th-M, 5th instar 36 h molting larvae; 6th-6 h through 6th-120 h, hours of the sixthth instar larvae; 5F, 5th instar feeding larvae; 5M, 5th instar molting larvae; 6F, sixth instar feeding larvae; MM, metamorphic molt; 6th-F, sixth instar feeding larvae; P0, pupae at day 1. **(c)** Relative quantification of the data in **(C)** using the Image Pro-Plus software. Bars indicate mean ± SD of three independent experiments. **(D)** qRT-PCR analysis of the expression levels of *ATG12* mRNA in the midgut after 20E induction (500 ng applied per 6th-6 h larva). **(E)** qRT-PCR analysis of the expression levels of *ATG12* in HaEpi cells after 20E treatment (5 µM) at the corresponding time points. Bars represent mean ± SD of three independent experiments. The data in **(D,E)** were subjected to Student’s *t*-test to indicate significant differences relative to the dimethyl sulfoxide (DMSO) controls at the corresponding time points. *p < 0.05; **p < 0.01.

A previous study indicated that ATG12 forms the ATG12–ATG5 conjugate in yeast (5). Therefore, we tested whether ATG12 and ATG5 formed a conjugate in an HaEpi and midgut. The anti-ATG12 antibodies recognized two bands from the lysate of HaEpi after DMSO and 20E treatments, with the 14 kDa band corresponding to the calculated molecular mass of free ATG12 (14.2 kDa) and the 45 kDa band corresponding to ATG12 (14.2 kDa) plus ATG5 (31 kDa), indicating that the anti-ATG12 antibody could recognize ATG12 and the ATG12–ATG5 conjugate. The anti-ATG12 antibodies also recognized these two bands in the larval midgut from sixth instar 24 h feeding larvae and 96 h MM larvae. There were lower levels of free ATG12 in both the sixth instar 24 h feeding larvae and 96 h MM larvae, and an increased level of the ATG12–ATG5 conjugate at the sixth instar 96 h larvae (Figure 1A), suggesting that ATG12 forms conjugate with ATG5 once it is expressed in the MM larval midgut.

To confirm that the 45-kDa band was indeed the ATG12–ATG5 conjugate, we used antibodies recognizing *H. armigera* ATG5 prepared in our previous work (19). The anti-ATG5 antibodies also detected the 45-kDa band (ATG12–ATG5 conjugate) in both HaEpi and midgut samples. In addition, full length ATG5 (31 kDa) and a very weak cleaved N-terminal ATG5 fragment (NtATG5, 25 kDa) were also detected by the anti-ATG5 antibodies (Figure 1B). These results confirmed that ATG5 forms a conjugate with ATG12, and that the anti-ATG5 antibodies specifically recognize ATG5 and the ATG12–ATG5 conjugate.

We next examined the variation in ATG12–ATG5 conjugate levels in greater detail in selected *H. armigera* tissues starting from the fifth instar feeding larval stage until pupation. The apparent abundance of the 45-kDa ATG12–ATG5 conjugate increased during the MM period at 72, 96, and 120 h of the sixth instar in the epidermis, midgut, and fat body, and decreased at pupation time (Figures 1C,c). This suggested that ATG12–ATG5 conjugate levels in these tissues are possibly regulated by 20E during metamorphosis, because the 20E titer can reach 4.87 µg/mL (about 10 µM) from 0.02 µg/ml in lepidopteran insect *Antheraea mylitta* at the molting and spinning stages (27). The highest titer in the hemolymph of *H. armigera* increased from 100 to 800 ng/mL, as assessed by radioimmunoassay (28). To examine whether ATG12 expression might be regulated by 20E at the mRNA level, we injected 20E into the hemocoel of 6th-6 h larvae. The qRT-PCR results showed that *ATG12* mRNA in the midgut was upregulated significantly at 12 h after 20E injection compared with larvae injected with the DMSO solvent. The upregulation lasted for at least 24 h post-injection (Figure 1D). Similarly, 20E-induced *ATG12* expression after 12 h and the effect lasted for at least 48 h in the HaEpi, compared with a control treated with the equivalent amount of DMSO solvent (Figure 1E). These results suggested that *ATG12* expression is stimulated by 20E.

### The Abundance of the ATG12–ATG5 Conjugate Is Increased by a Low Concentration and a Short Period of Treatment of 20E, and Repressed by a High Concentration and a Long Period of Treatment of 20E

**Figure 2:**
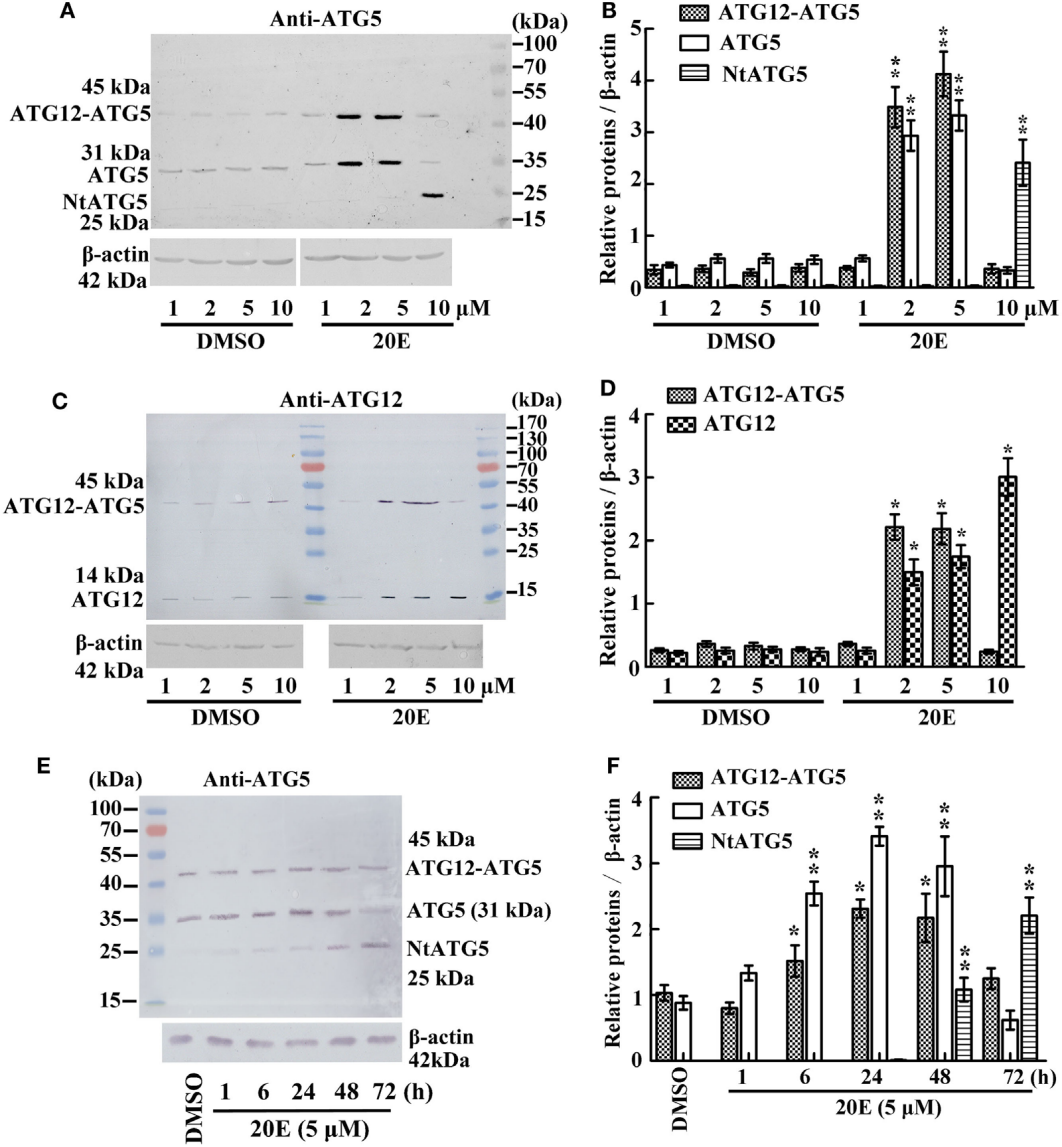
Regulation of 20-hydroxyecdysone (20E) on ATG12–ATG5 conjugate levels by western blot analysis. **(A)** Anti-ATG5 antibody detecting the levels of the ATG12–ATG5 conjugate in *Helicoverpa armigera* epidermis cell line (HaEpi) cells under 20E treatment (24 h) at the indicated concentration. **(B)** Statistical analysis of **(A)**. **(C)** Anti-ATG12 antibody detecting the levels of the ATG12–ATG5 conjugate in HaEpi cells as the condition as **(A)**. **(D)** Statistical analysis of **(C)**. **(E)** Anti-ATG5 antibody detecting the levels of the ATG12–ATG5 conjugate in HaEpi cells under 20E treatment (5 µM) at the indicated time. **(F)** Statistical analysis of **(E)**. Bars represent mean ± SD of three independent experiments. Asterisks indicate significant differences determined by Student’s *t*-test: *p < 0.05; **p < 0.01 in all of the statistical analysis.

To examine whether 20E regulates the ATG12–ATG5 conjugate levels, we determined the variation in the ATG12–ATG5 conjugate levels under 20E induction at various concentration in HaEpi cells. The anti-ATG5 antibody detected a marked increase in both ATG5 (31 kDa) and the ATG12–ATG5 conjugate (45 kDa) after induction by 2–5 µM 20E. However, ATG5 (31 kDa) and ATG12–ATG5 conjugate (45 kDa) levels decreased, but NtATG5 (25 kDa) increased significantly, under a high concentration of 20E induction (10 µM) compared with the control (DMSO) (Figures 2A,B). In turn, the anti-ATG12 antibody also detected the increase of ATG12 (14 kDa) and the ATG12–ATG5 conjugate (45 kDa) levels after induction using low concentrations of 20E (2–5 µM). However, the ATG12–ATG5 conjugate level decreased significantly under a higher concentration of 20E (10 µM) induction, but ATG12 level was not decreased simultaneously (Figures 2C,D). These data confirmed that a lower concentration of 20E promotes ATG12 expression and ATG12–ATG5 conjugation, but a higher concentration of 20E represses ATG12–ATG5 conjugation.

Different periods of 20E treatment were examined to analyze the effect of 20E on ATG12–ATG5 conjugate levels. Both ATG5 (31 kDa) and ATG12–ATG5 conjugate (45 kDa) levels increased from 1 to 48 h under 5 µM 20E induction. However, ATG5 and ATG12–ATG5 conjugate levels decreased, but NtATG5 (25 kDa) increased significantly, after a longer period of 20E induction (72 h) (Figures 2E,F). These data suggested that a shorter period of 20E treatment induces ATG5 expression and ATG12–ATG5 conjugate formation, but a longer period of 20E treatment decreases ATG5 and ATG12–ATG5 conjugate levels by causing the cleavage of ATG5 to NtATG5.

**Figure 3:**
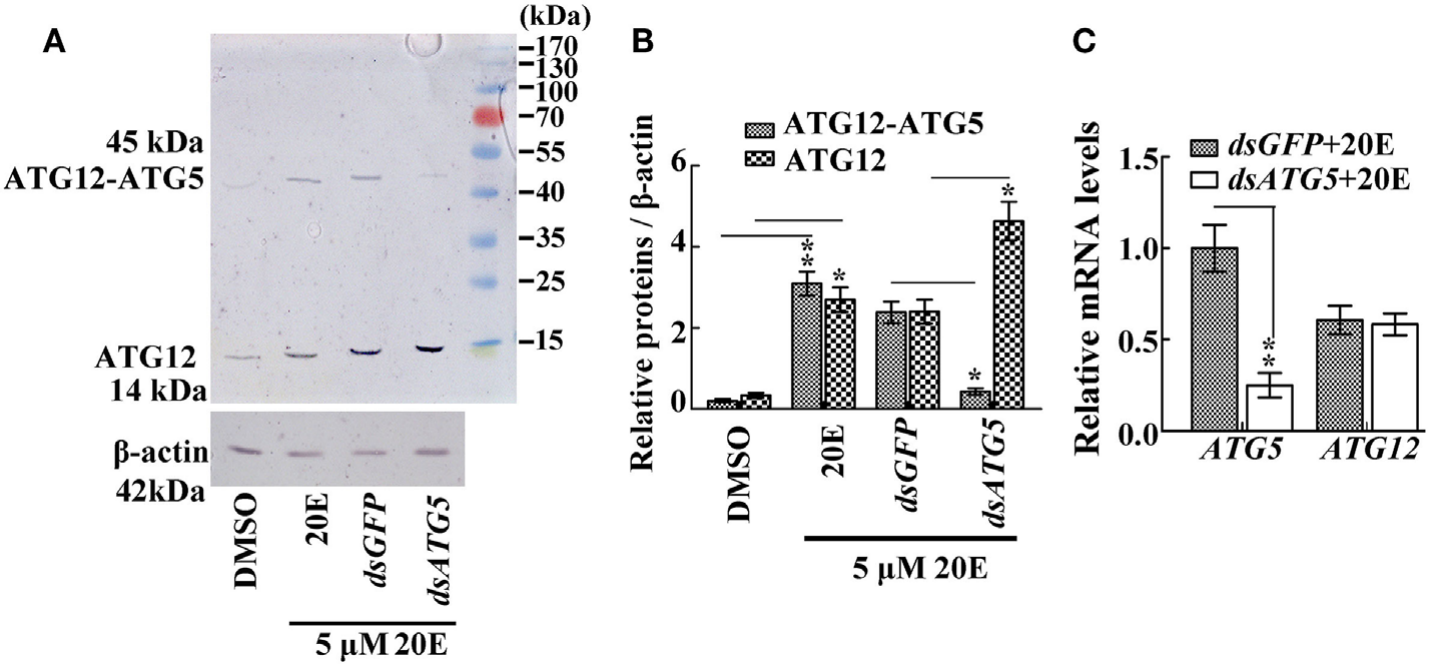
The effect of ATG5 knockdown to ATG12–ATG5 conjugate. **(A)** ATG12–ATG5 conjugate levels and free ATG12 protein after knockdown of *ATG5* and 20E treatment for 24 h. **(B)** Statistical analysis of **(A)**. **(C)** Expression levels of the *ATG12* mRNA after *ATG5* knockdown and 20E treatment for 24 h. β-actin served as an internal reference for all the experiments. Bars represent mean ± SD of three independent experiments. Asterisks indicate significant differences determined by Student’s *t*-test: *p < 0.05; **p < 0.01 in all of the statistical analysis.

To prove the key role of ATG5 in maintaining the ATG12–ATG5 conjugate, *ATG5* was knocked down in HaEpi cells. Immunoblotting showed that, as expected, *ATG5* depletion reduced the amount of the ATG12–ATG5 conjugate, while the relative amount of unconjugated ATG12 increased (Figures 3A,B). Meanwhile, *ATG5* knockdown had no effect on *ATG12* expression at the mRNA level (Figure 3C). These data confirmed that the ATG5 level determines ATG12–ATG5 conjugate levels.

### ATG12 Was Located in the Larval Midgut during Metamorphosis

**Figure 4:**
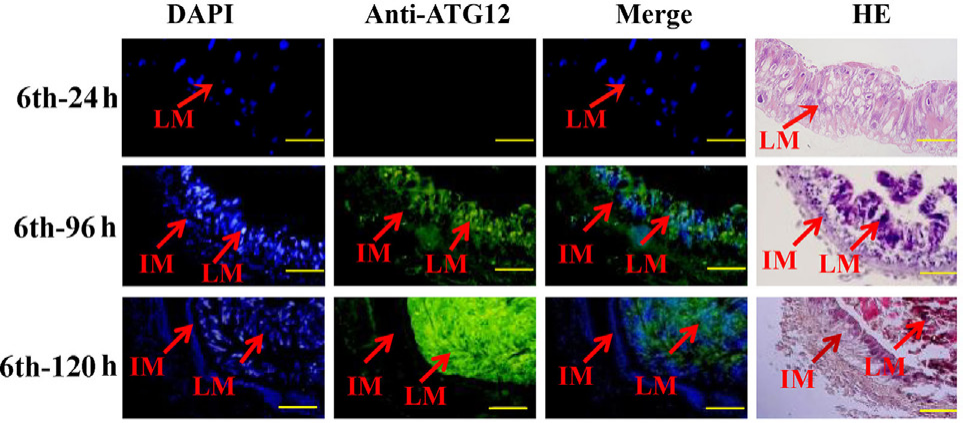
Localization of ATG12 in the midgut. The tissues dissected from the sixth instar larvae at the indicated time points. Stained with 6-diamidino-2-phenylindole (DAPI) and with the anti-ATG12 antibody followed by an Alexa 488-labeled secondary antibody, or with hematoxylin and eosin (HE). LM, larval midgut; IM, imaginal midgut. Scale bars = 50 µm.

The location of ATG12 was examined by immunohistochemistry using anti-ATG12 antibodies to address its role in midgut PCD during metamorphosis. The results showed that anti-ATG12 antibodies detected an intense signal in the larval midgut cells from the 6th-96 h and 6th-120 h wandering larvae. In contrast, a less intense signal was detected from the 6th-24 h feeding larval midgut or the imaginal midgut cells (Figure 4), suggesting ATG12 was localized in the larval midgut.

### *ATG12* Knockdown Delays Pupation and Blocks Midgut PCD

**Figure 5:**
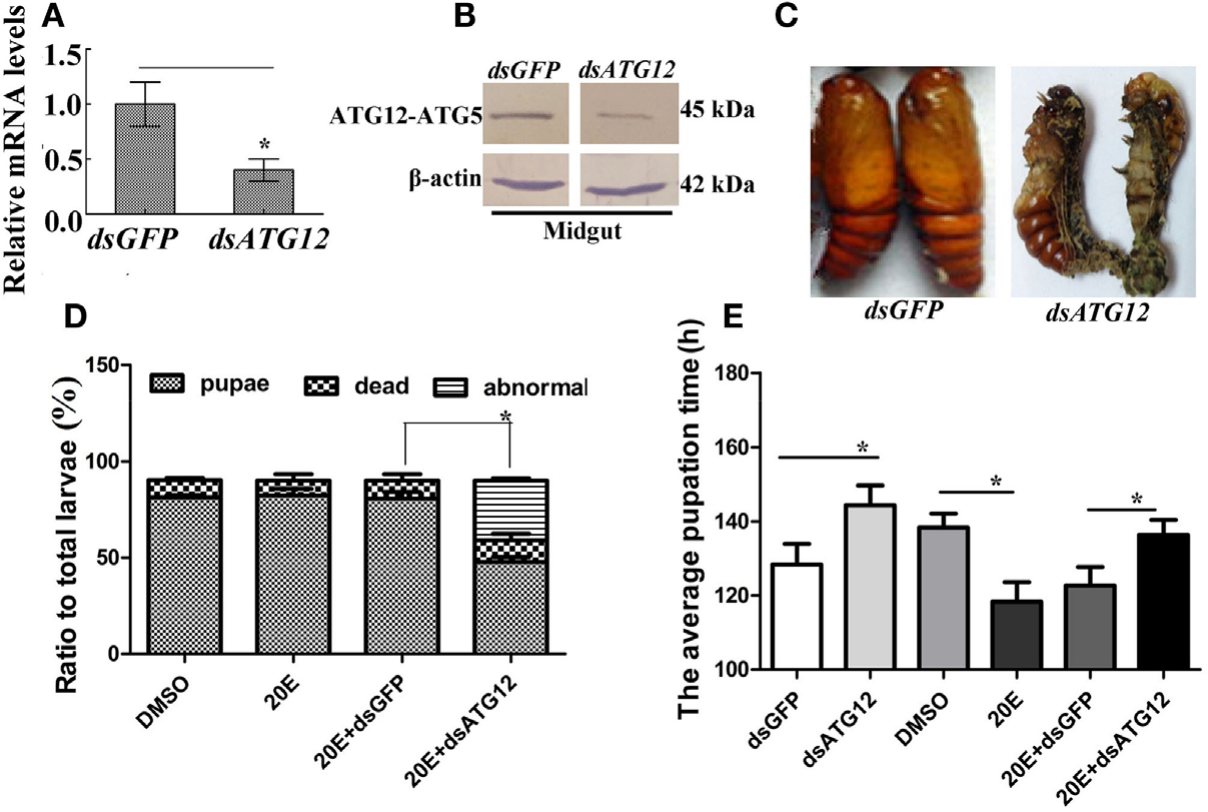
*ATG12* knockdown delayed 20-hydroxyecdysone (20E)-induced pupation. One µg of double-stranded RNA (dsRNA) and 500 ng 20E were injected into sixth instar 6 h larvae; 3 groups and 30 larvae in each group. After 24 h, 1 µg of dsRNA was injected again. **(A,B)** qRT-PCR and immunoblotting indicate the efficiency of *ATG12* RNA interference at the mRNA and protein levels, respectively. **(C)** The phenotype of insects after *ATG12* knockdown. **(D)** Statistical analysis of the phenotype shown in **(C)**. **(E)** Statistical analysis of pupation time of 50% larvae (P_50_) after injection of *dsRNA*. Bars represent mean ± SD of three independent experiments. Asterisks indicate significant differences determined by Student’s *t*-test: *p < 0.05; **p < 0.01.

To investigate the function of ATG12 in insect pupation, *ATG12* was knocked down by injecting the larvae with dsRNA. qRT-PCR and immunoblotting showed that *ATG12* was knocked down systemically in the larval midgut (Figures 5A,B). *ATG12* knockdown resulted abnormal pupation (Figure 5C). Compared with *dsGFP* injection, 33% of the larvae formed abnormal pupae and many of the abnormal pupae died (Figure 5D). Although 50% of the larvae survived and pupated after *dsATG12* injection, the pupation time was delayed to 145 h, which was 12 h longer, compared with the *dsGFP*-injected larvae (133 h). In addition, 20E injection caused larval pupation at 118 h, which was 20 h earlier compared with that in the DMSO-injected larvae (138 h). However, after *dsATG12* injection, 20E could not promote earlier pupation, but delayed this 20E-stimulated pupation by 16 h, increasing the pupation time from 120 to 136 h, compared with *dsGFP* plus 20E (Figure 5E). These results suggested that ATG12 is critical for the 20E-dependent promotion of pupation.

**Figure 6:**
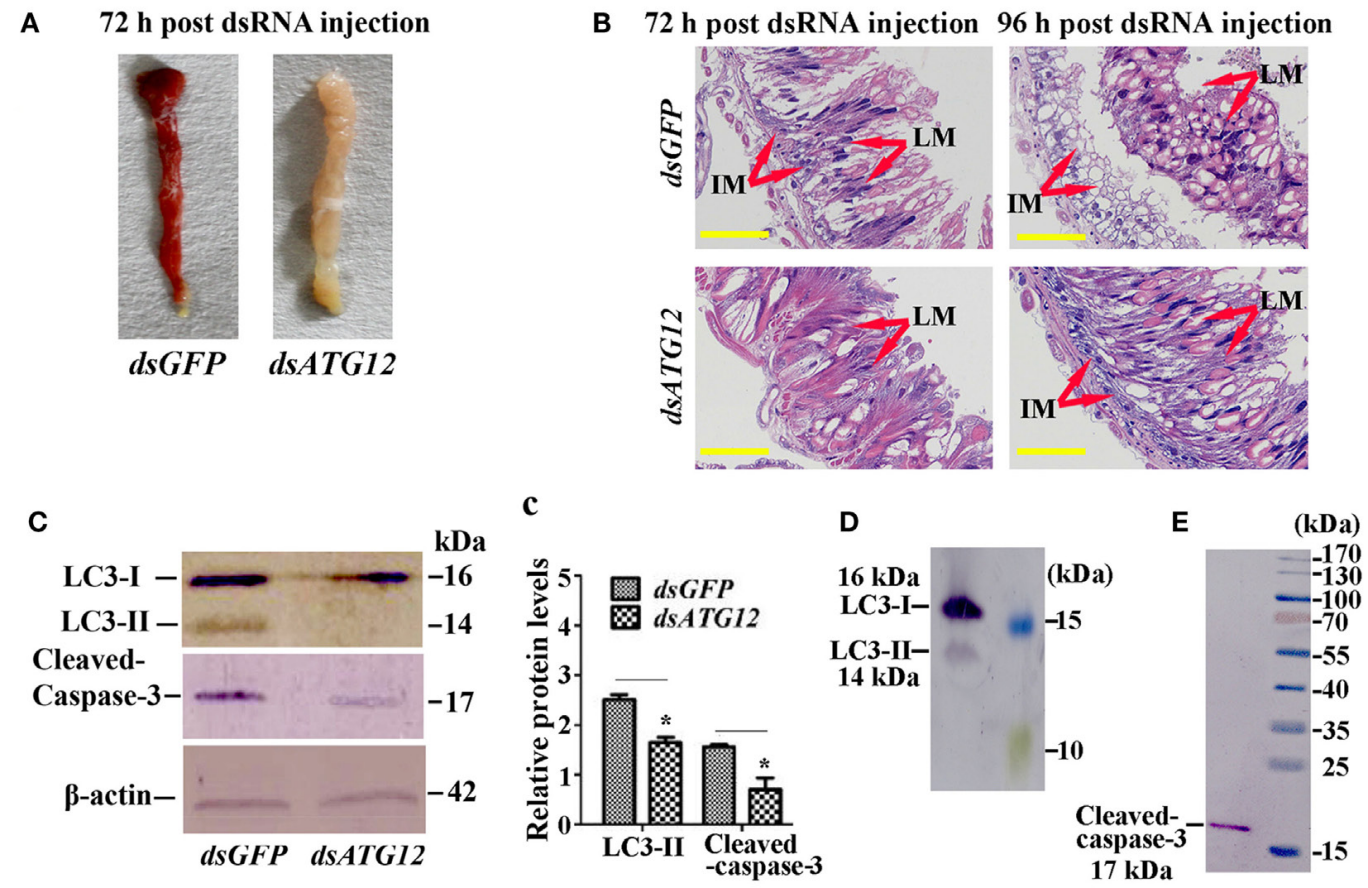
Knockdown of *ATG12* delayed midgut programed cell death. **(A)** The phenotype of the midgut 72 h after *dsATG12* (1 µg) injection to sixth instar 6 h larvae. **(B)** Hematoxylin and eosin staining shows the midgut structure 72 and 96 h after *dsGFP* and *dsATG12* injection, respectively. **(C)** Levels of the indicated proteins in the midgut after *ATG12* knockdown using antibodies against *H. armigera* LC3 and β-actin; cleaved Caspase-3 was detected with the “anti-mammalian” Caspase-3 antibody. **(c)** Statistical analysis of the data in **(C)**. Bars represent mean ± SD of three independent experiments; significance of differences determined by Student’s *t*-test: *p < 0.05; **p < 0.01. **(D,E)** The specificity of the antibodies and molecular weight of LC3 and Caspase-3. The standard molecular masses of the proteins are same as in Figure 1A.

To examine the role of ATG12 in midgut PCD, we observed midgut structures after *dsATG12* injection in 6th-6 h larvae. The midgut developed a red color, also known as the yellow body (29), which is a typical characteristic of midgut PCD (30), at 72 h after *dsGFP* injection. However, the midgut remained milky white, which is typical of a feeding midgut, after *dsATG12* injection (Figure 6A). During midgut PCD, the larval midgut is separated from imaginal midgut and larval midgut undergoes PCD (29). Immunohistochemical analysis showed that after *dsATG12* injection, the larval midgut did not separate from the imaginal midgut at 96 h. In contrast, the larval midgut separated from the imaginal midgut at 96 h after *dsGFP* injection (Figure 6B). These results suggested that ATG12 is required for larval midgut PCD.

To address the mechanism whereby *ATG12* knockdown represses midgut PCD, we examined the ATG levels of LC3-II (ATG8–PE), and cleavage of apoptosis-associated protein caspase-3, with LC3-II indicating autophagy and caspase-3 cleavage indicating apoptosis. The results showed that at 72 h after *dsATG12* injection, the level of LC3-II, the autophagic indicator, was significantly lower relative to that in *dsGFP-*injected animals. The level of cleaved caspase-3, the apoptosis indicator, was also reduced significantly (Figures 6C,c). The specificity of the antibodies against LC3-II and caspase-3 were confirmed by western blotting (Figures 6D,E). These results suggested that ATG12 is involved in both autophagy and apoptosis during midgut PCD.

### *ATG12* Knockdown Represses Autophagosome Formation and Caspase-3 Activity

**Figure 7:**
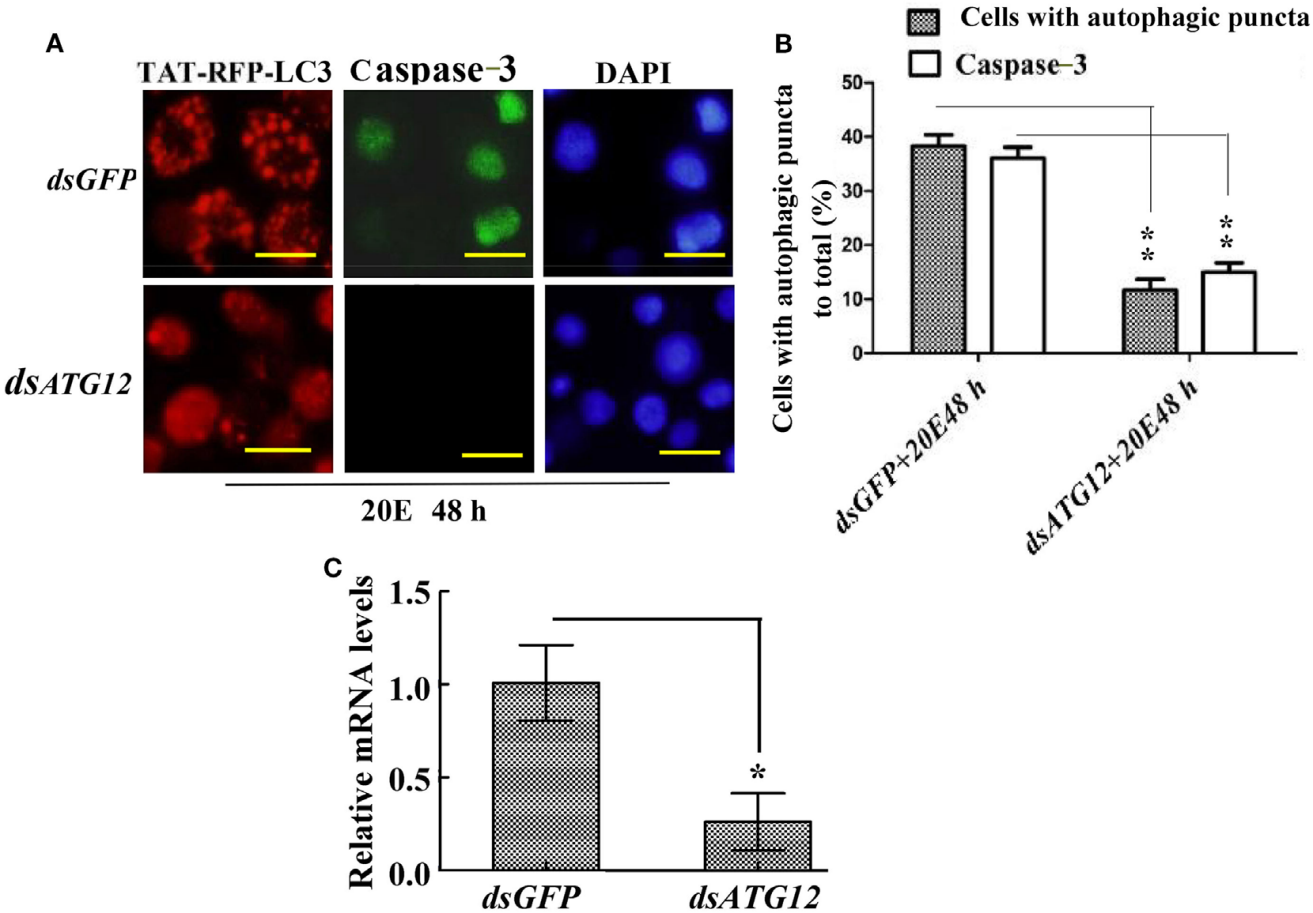
Knockdown of *ATG12* repressed autophagy and apoptosis in *Helicoverpa armigera* epidermis cell line cells. **(A)** *ATG12* RNA interference (RNAi) repressed autophagosome formation and Caspase-3 activity. Cells were exposed to 5 µM 20E and either *GFP* (control) or *ATG12* double-stranded RNA for 48 h. Red fluorescence indicates the TAT-RFP-LC3 protein, a cell-penetrating peptide (TAT) fused to the LC3 protein; green fluorescence indicates Caspase-3 activity detected by the Nuclear View Caspase-3 assay kit. Scale bars = 50 µm. **(B)** Statistical analysis of the effects of *ATG12* RNAi on autophagosome formation and Caspase-3 in **(A)**. **(C)** qRT-PCR analysis of the knockdown efficiency of *ATG12*. Bars represent mean ± SD of three independent experiments; significance of differences determined by Student’s *t-*test: *p < 0.05; **p < 0.01 in all the statistics.

To confirm the role of ATG12 in autophagy and apoptosis, we examined the involvement of ATG12 in autophagy and apoptosis in HaEpi cells. We constructed a red fluorescence protein-marked LC3 protein by fusing it with the cell-penetrating peptide TAT (TAT-RFP-LC3), with the red fluorescence protein indicating LC3, and the TAT penetrating peptide allows the protein to enter into cells. The TAT-RFP-LC3 protein accumulated as puncta in *dsGFP*-treated cells, indicating autophagosome formation; however, the TAT-RFP-LC3 protein did not accumulated as puncta, but existed uniformly in *dsATG12*-treated cells, indicating no autophagosome formation in *dsATG12*-treated cells. Meanwhile, caspase-3 activity was detected in *dsGFP*-treated cells, but not in *dsATG12*-treated cells (Figure 7A). Statistical analysis indicated significant differences in the number of autophagosome puncta and caspase-3 activity between the *dsGFP* and *dsATG12*-treated cells (Figure 7B). qRT-PCR confirmed the successful knockdown of *ATG12* in HaEpi cells (Figure 7C). These data suggested that ATG12 is involved in both autophagy and apoptosis.

## Discussion

The ATG12–ATG5 conjugate is required for LC3 lipidation for final autophagy (4). However, whether steroid hormones regulate ATG12–ATG5 conjugate formation was unknown until now. In the present study, we present evidence that the steroid hormone 20E promotes ATG12–ATG5 conjugate formation under low concentrations and for a short period, but inhibits ATG12–ATG5 conjugate formation at a high concentration and for a long period. In addition, ATG12 is necessary by autophagy and apoptosis.

### 20E Regulates ATG12–ATG5 Conjugate Levels in a Concentration and Time-Dependent Manner

As one of the core ATG proteins, ATG12 plays an important role in autophagy by forming a complex with ATG5 and ATG16 to accelerate the formation of ATG8–PE (LC3-II) and autophagy precursors, with a double membrane structure (31). The human ATG12–ATG5 conjugate appears as a single band with a molecular mass of about 52 kDa (4). Our study revealed that the ATG12–ATG5 conjugate is abundant in the midgut during the wandering stages from sixth instar 72–120 h, but decreases at the pupation stage, indicating the occurrence of autophagy at an earlier metamorphic stage, which had stopped by the pupation stage. This finding correlated with the observation that autophagy occurs at an earlier stage of MM, and apoptosis occurs at the later stage of MM in lepidopteran insects (16). The 20E titer is higher at the wandering stages in lepidopteran insects (13); therefore, we hypothesized that the level of the ATG12–ATG5 conjugate is increased *via* 20E induction. However, an interesting finding from our study is that 20E induction increases ATG12–ATG5 conjugate only at low concentration (2–5 µM) and for a short period (1–48 h), but inhibits ATG12–ATG5 conjugate formation at a high concentration (10 µM) and for a longer period (72 h). We observed that higher 20E concentration (10 µM) and longer time (72 h) treatment induced cleavage of the full length ATG5 to NtATG5, and inhibited the formation of the ATG12–ATG5 conjugate.

ATG5 cleavage to NtATG5 is part of the mechanism of autophagy switching to apoptosis. ATG5 is cleaved by calcium-dependent cysteine proteases (calpains) to NtATG5, which binds to mitochondria to release cytochrome C, triggering the activation of caspases and apoptosis (32). Higher 20E concentrations induce higher calcium levels and the transformation of ATG5 to NtATG5 to switch autophagy to apoptosis in the midgut PCD (19). Therefore, 20E triggers autophagy at lower concentration and transform autophagy to apoptosis at higher concentration by the cleavage of ATG5 to NtATG5.

### 20E Regulates Autophagy and Apoptosis Dependent on Concentration

20-Hydroxyecdysone promotes both autophagic and apoptotic genes expression, such as in the fat body of *B. mori* (17, 18). In the *Drosophila* and *Bombyx* fat body, overexpression of a dominant-negative mutant of the 20E receptor EcR suppressed 20E-induced autophagy (18, 33). The promoter of *ATG1* contains an *EcR* response element (*EcRE*) and deletion of the *EcRE* inhibited 20E-induced autophagy in the *Bombyx* fat body (18).

20-Hydroxyecdysone plays a critical role in insect development, particularly in PCD during the developmental transition from larvae to adults (34). The 20E titer increases during the molting and metamorphic stages in *Drosophila* (35) and in Lepidoptera (13), reaching 900 ng/mL in the *Bombyx* wing disk during metamorphosis (36). The highest 20E titer was recorded as 4.87 µg/mL in the hemolymph (about 10 µM) in *A. mylitta* (lepidoptera: Saturniidae) at the molting stage (27). The 20E titer increased to 800 ng/mL from a much lower titer in the hemolymph in *H. armigera* (28). We observed that 20E promotes ATG12 and ATG5 expression and ATG12–ATG5 conjugate formation; however, a higher concentration and longer time of 20E treatment induced the cleavage of ATG5 to NtATG5 and decreased the ATG12–ATG5 conjugate levels. These results confirmed that 20E promotes autophagy at a lower concentration and shorter time, but switches autophagy to apoptosis by inducing ATG5 cleavage to NtATG5 at a higher concentration and longer exposure.

### Autophagy Is Essential for Apoptosis in the Midgut of *H. armigera*

Both autophagy and apoptosis are observed during *Drosophila* midgut PCD (37). Apoptosis involves nuclear condensation, membrane blebbing, DNA fragmentation, and activation of caspases; whereas autophagy is accompanied by the formation of acidic autophagic puncta in the cytoplasm (38). One study suggested that autophagy is essential for midgut cell death in *Drosophila* (39); however, autophagy and apoptosis occur sequentially during midgut PCD of *B. mori* (40). In the human liver, autophagy also precedes apoptosis, and is required for the occurrence of apoptosis (41). In *H. armigera*, apoptotic midgut PCD has been observed (11). Our previous study showed that an inhibitor of autophagy, 3-MA, or knockdown of *ATG12* repressed autophagy and apoptosis. In addition, after knockdown of *ATG5*, autophagy and apoptosis are inhibited in the midgut, and 20E promoted ATG5 cleavage to NtATG5, which mediates the transformation of autophagy to apoptosis (19). In the present study, our data showed that knockdown of *ATG12* in larvae also delayed pupation and midgut PCD. Meanwhile, LC3-II levels and activated caspase-3 were also decreased by *ATG12* knockdown. This suggested that both autophagy and apoptosis are involved in midgut PCD in *H. armigera*, and that autophagy is essential for the occurrence of apoptosis. *ATG12* knockdown in the HaEpi cells not only repressed autophagosome formation, but also repressed caspase-3 activity. Therefore, our data showed that during PCD of the midgut in *H. armigera*, autophagy is essential for apoptosis, and that ATG12 plays a role in both processes.

## Conclusion

**Figure 8:**
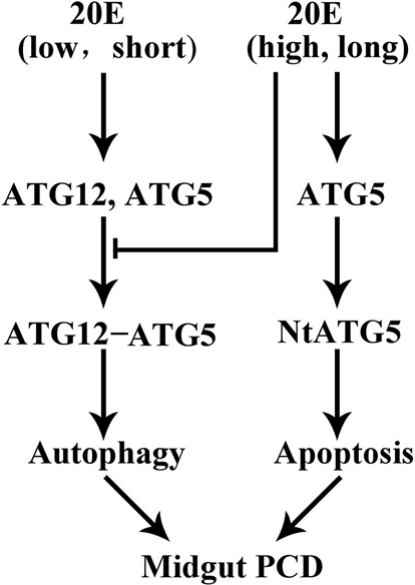
A model for 20-hydroxyecdysone (20E) regulation on ATG12–ATG5 conjugate formation and midgut programed cell death (PCD). 20E promotes ATG12 and ATG5 expression and ATG12–ATG5 conjugate formation at a lower concentration, shorter period, thus results in autophagy. Whereas, 20E inhibits ATG12–ATG5 conjugate formation by inducing the cleavage of ATG5 to NtATG5 at a higher concentration, longer period, thus causes apoptosis during midgut PCD.

Based on the above data, we conceived a model to explain the role of ATG12 in autophagy and apoptosis under 20E regulation (Figure 8). A low concentration and short period of treatment by 20E promote the expression of ATG12 and ATG5 and the conjugation ATG12 and ATG5, which subsequently promotes autophagy. A high concentration and a long period of treatment by 20E inhibit ATG12–ATG5 conjugation because ATG5 is cleaved to NtATG5 for apoptosis. ATG12 is necessary for autophagy and apoptosis in the insect midgut PCD.

## Author Contributions

Y-BL prepared the antibodies against *H. armigera* ATG5 and performed most of the experiments. TY prepared the antibodies against *H. armigera* ATG12 and performed some of the experiments. J-XW and X-FZ conceived and coordinated the study and edited the paper. All authors reviewed the results and approved the final version of the manuscript. All co-authors have checked and confirmed their contribution statement.

## Conflict of Interest Statement

The authors declare that they have no conflicts of interest with the contents of this article.

## Footnotes

^1^http://web.expasy.org/translate/.

^2^http://quantity-one.software.informer.com/.

^3^https://www.genetargetsolutions.com.au/product/nucview-488-caspase-3-assay-kit-live-cells-biotium/.

## Accession Number

GenBank accession number of ATG12: KY217730 (X-FZ).
